# Outcome of 129 Pregnancies in Polycythemia Vera Patients: A Report of the European LeukemiaNET

**DOI:** 10.1097/HS9.0000000000000882

**Published:** 2023-05-02

**Authors:** Kai Wille, Maja Brouka, Johannes Bernhardt, Axel Rüfer, Emilia Niculescu-Mizil, Mirjana Gotic, Susanne Isfort, Steffen Koschmieder, Tiziano Barbui, Parvis Sadjadian, Tatjana Becker, Vera Kolatzki, Raphael Meixner, Hannah Marchi, Christiane Fuchs, Frank Stegelmann, Konstanze Döhner, Jean-Jacques Kiladjian, Martin Griesshammer

**Affiliations:** 1 University Clinic for Hematology, Oncology, Hemostaseology and Palliative Care, Johannes Wesling Medical Center Minden, University of Bochum, Germany; 2 Luzerner Kantonsspital, Division of Hematology, Luzern, Switzerland; 3 Provita Diagnosis and Treatment Center, Bucharest, Romania; 4 Clinic for Hematology Clinical Center of Serbia, Medical Faculty University of Belgrade, Serbia; 5 Department of Medicine (Hematology, Oncology, Hemostaseology and SCT), Faculty of Medicine, RWTH Aachen University, Germany; 6 Center of Integrated Oncology Aachen Bonn Cologne Düsseldorf (CIO ABCD), Germany; 7 Research Foundation, Papa Giovanni XXIII Hospital, Bergamo, Italy; 8 Core Facility Statistical Consulting, Helmholtz Zentrum München, Munich, Germany; 9 Faculty of Business Administration and Economics, Bielefeld University, Germany; 10 Department of Internal Medicine III, University Hospital of Ulm, Germany; 11 Université Paris Cité, AP-HP, Hôpital Saint-Louis, Centre d’Investigations Cliniques, Paris, France

Pregnancies in women with polycythemia vera (PV) are rare events due to the low disease incidence and prevalence,^1^ especially in women of childbearing age. However, about 15% of PV patients are diagnosed before the age of 40.^1,2^ In PV pregnancies, the high risk of miscarriage and maternal thrombosis are the main challenges for the treating physicians. To date, information on only about 200 PV pregnancies have been published, and a few studies^3–6^ suggest that administration of PV-specific therapies such as acetylsalicylic acid (ASA), low molecular weight heparin (LMWH), and/or interferon (IFN)-alpha during pregnancy may improve live birth rate.

To obtain data on the impact of antenatal PV-specific therapy on live birth rates and to investigate the effects of different variables on pregnancy outcome and maternal complications, a retrospective analysis of 129 PV pregnancies was conducted as part of the European LeukemiaNET “MPN Pregnancy Project.” Eligibility criteria include women with PV and at least 1 PV-associated pregnancy defined as pregnancy that began 2 years before PV diagnosis or at any time thereafter. The diagnosis of PV was made according to the diagnostic criteria of the World Health Organization (WHO) classification valid at the time of diagnosis. The main objective of the analysis was to determine the live birth rate and pregnancy-related complications and to assess the impact of the different PV-specific therapies on the course of pregnancy. The use of either ASA and/or LMWH in prophylactic dosage and/or IFN was considered as PV-specific therapy. Patients gave their written consent for data collection and the institutional review boards of all centers approved the study.

A total of 129 PV pregnancies in 69 patients from 11 European centers were included. Pregnancies were distributed as follows: “Johannes Wesling University Hospital,” Minden (Germany): n = 62 (data from 41/62 pregnancies had already been published^7^); “Hôpital Saint-Louis” (AP-HP, Université Paris Cité, France): n = 36; University hospital Ulm (Germany): n = 12; “Papa Giovanni XXIII” hospital, Bergamo (Italy): n = 5; “Luzerner Kantonsspital” (Switzerland): n = 5; Medical Faculty University of Belgrade (Serbia): n = 3; University Clinic of Bucharest (Romania): n = 2; “Hospital Belle Isle,” Metz (France): n = 2; University clinic Aachen (Germany): n = 1; “Kantonsspital Baselland” (Switzerland): n = 1. The 129 pregnancies were recorded between 1986 and 2022. Pregnancy outcome was defined as spontaneous abortion (pregnancy loss before or in the 20th week), stillbirth (intrauterine death after 20 weeks without signs of life at birth), preterm delivery (live birth with a birth weight below 2.5 kg or birth between weeks 24 and 37), or full-term normal delivery (live birth after 37 weeks, including overdue deliveries).^7,8^ The total follow-up time was calculated by summing up the periods from the onset of pregnancy, if it started before PV diagnosis, or the PV diagnosis (whichever came first) until the last contact with each center. Maternal pregnancy complications were defined as any adverse event occurring during pregnancy or within 6 weeks postpartum.

The median age of the 69 patients was 33.9 years (range, 21.4–42.6) at delivery, and the median number of pregnancies per patient was 2 (range, 1–5). The median follow-up time of all 129 pregnancies was 10.6 years overall (range, 1.5–33.1). Delivery occurred at a median of 38 weeks of gestation (range, 4–42). A total of 23 pregnancies (17.8%) had their delivery date before (n = 20) or at the time (n = 3) of PV diagnosis. In 106 (82.2%) pregnancies, delivery occurred after PV diagnosis. Delivery by cesarean section was performed in 27 pregnancies (20.9%). Live births occurred in 68.2% (88/129) of pregnancies, with full-term normal deliveries accounting for 50.4% (65/129). Premature delivery occurred in 17.8% (23/129). The miscarriage rate was 31.8% (41/129), with 24.8% of pregnancies (32/129) ending in spontaneous abortion and 7.0% (9/129) in stillbirth.

PV-specific therapy was administered in 87 of 129 (67.4%) pregnancies and resulted in live births in 78.2% (68/87). In contrast, live birth rate in the 42 pregnancies without PV-specific therapy was 47.6% (20/42). Among the 23 (17.8%) pregnancies with delivery before/at the time of PV diagnosis, live birth rate was 47.8% (11/23). The most commonly used PV-specific therapy combination was ASA with LMWH, applied in 29.5% of all pregnancies (38/129). ASA monotherapy was used in 18.6% (24/129) and LMWH monotherapy in 11 pregnancies (8.5%). IFN was administered during 14 pregnancies (10.9%). In 2 of these 14 cases, IFN was used as a monotherapy, while IFN was combined with ASA and LMWH in 8, with ASA alone in 3, and with LMWH in 1 pregnancy, respectively.

Regarding statistical methods, 55.1% (38/69) of patients had >1 pregnancy, leading to dependent observations. Therefore, logistic regression in the class of generalized linear mixed models (GLMM) was used to model the effects of multiple variables on the binary variable pregnancy outcome. The following independent variables were defined: age at pregnancy establishment, delivery before or at the time of PV diagnosis, administration of a PV-specific therapy during pregnancy with ASA monotherapy, or LMWH monotherapy, or ASA in combination with LMWH, or IFN (monotherapy or in combination with ASA and/or LMWH). The estimated odds ratios for the covariates are presented in Figure 1 and in the Suppl. Table S1. According to this analysis, administration of ASA in combination with LMWH (*P* = 0.001) or IFN (alone or in combination with ASA and/or LMWH) (*P* = 0.023) was associated with a statistically significant lower risk of miscarriage than standard antenatal care.

**Figure 1.**
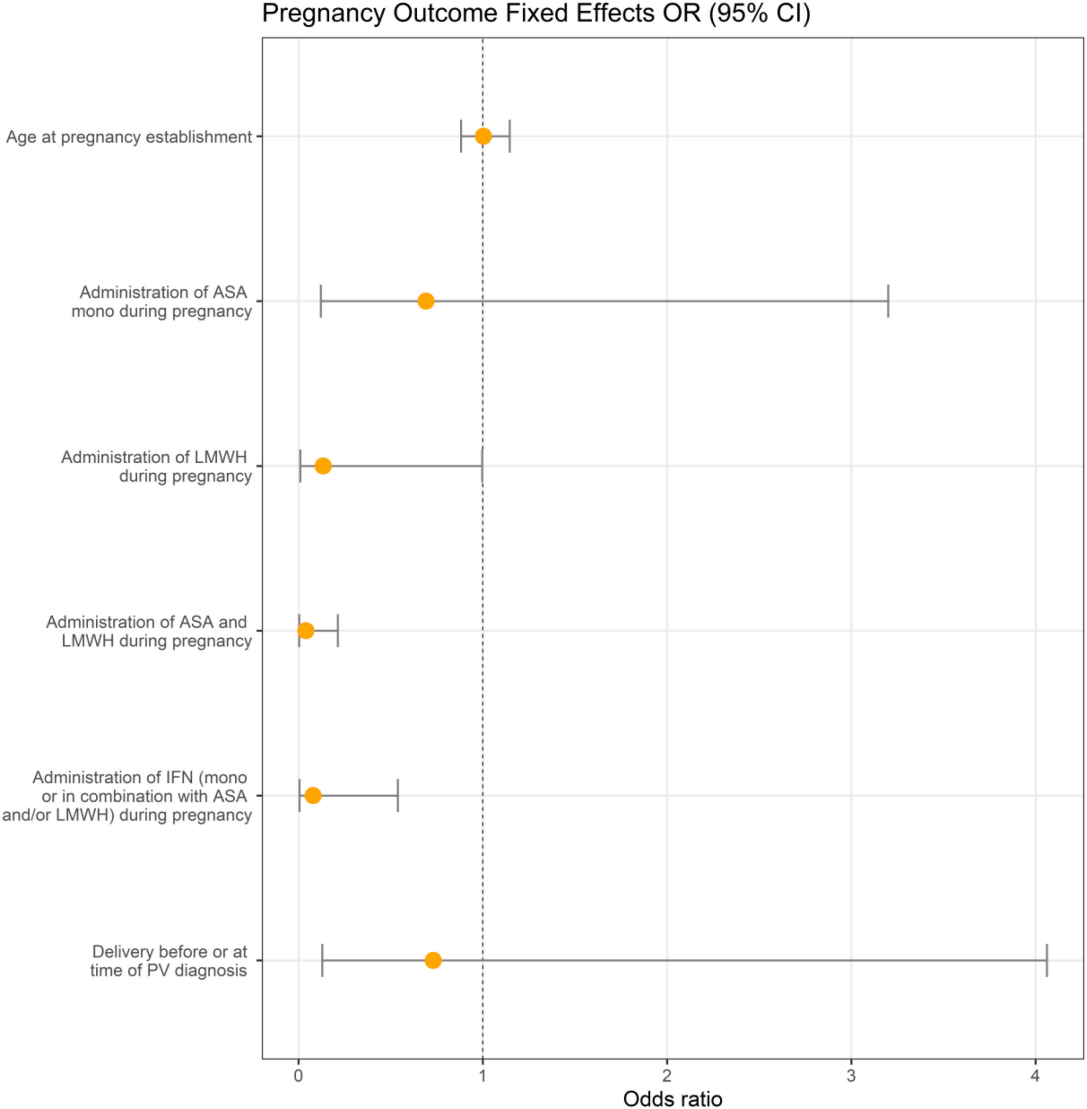
**Graphical representation of the application of GLMMs of the 129 PV pregnancies in relation to the outcome of the OR (95% CI ).** Administration of ASA/LMWH (*P* = 0.001) and IFN (*P* = 0.023) was statistically significantly associated with a lower risk of miscarriage than standard antenatal care. ASA = acetylsalicylic acid; CI = confidence interval; IFN = interferon; LMWH = low molecular weight heparin; PV = polycythemia vera; OR = odds ratio; GLMMs = generalized linear mixed models.

Maternal complications occurred in 29 of the 129 pregnancies (22.5%). Bleeding was the most common (n = 20), followed by 5 pre-eclampsia and 4 thromboembolic complications. Of all 20 bleeding complications, 4 were classified as major bleeding (20%) and 16 as minor bleedings (80%). Five cases of pre-eclampsia occurred in 5 (3.9%) pregnancies. No HELLP syndrome was reported. Of the 4 pregnancy-related thromboembolic complications (4/129, 3.1%), 3 were deep vein thromboses and 1 was a Budd-Chiari syndrome. Maternal pregnancy complications were comparable between women who received PV-specific therapy (23.9%, 21/88 pregnancies) and women without PV-specific therapy (19.5%, 8/41 pregnancies). No fatal maternal or fetal complications occurred.

To analyze covariates that might influence the risk for maternal complications, again a logistic GLMM was used with the previously established independent variables. None of these variables had a significant impact on maternal complications (presented in Figure 2 and in the Suppl. Table S2).

**Figure 2.**
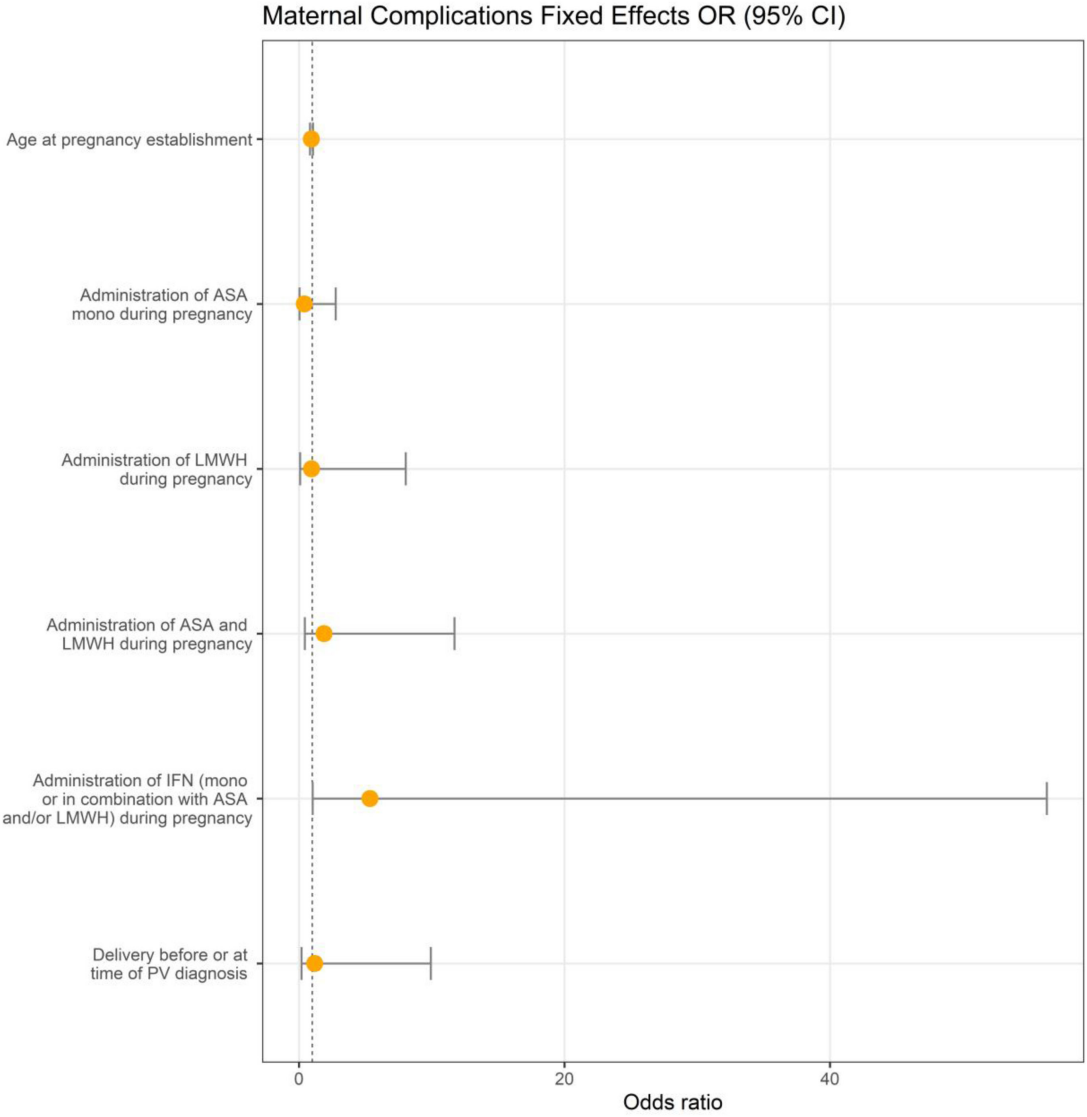
**Graphical representation of GLMMs of the 129 PV pregnancies in terms of maternal complications**. No significant association was found between these variables and maternal complications. CI = confidence interval; GLMMs = generalized linear mixed models; PV = polycythemia vera.

To date, the precise impact of PV-specific therapy on maternal complications or miscarriages in PV pregnancies is unknown because of the rare occurrence of the disease in women under 40 years of age and the small number of published cases.^9,10^ So far, most of the reported courses (159 PV pregnancies; none of our 129 cases) were included in the meta-analysis of Maze et al.^4^ The total live birth rate was only 66.7%. For comparison, 85% is expected for the general population.^11^ Of note, a recently published Swedish study^12^ using a population-based dataset showed significant better pregnancy outcomes (only 2 stillbirths and 1 neonatal death) in 342 MPN pregnancies (including 43 PV cases). However, only pregnancies that had reached 22 weeks of gestation were included in this analysis, which excludes all spontaneous abortions (78.0% of all miscarriages in our study were spontaneous abortions). Another study from the United Kingdom,^13^ which prospectively investigated 58 MPN pregnancies, showed also an impressive live birth rate (100%), but only 5 of 58 (9%) included cases were PV pregnancies. The live birth rate of our cohort was 68.2% (comparable to the analysis by Maze et al^4^) and administration of PV-specific therapy markedly improved this live birth rate to 78.2%. Specifically, administration of ASA in combination with LMWH or of IFN (alone or in combination with ASA and/or LMWH) were associated with a reduced risk of miscarriage as independent cofactors. This benefit could not be demonstrated for the use of ASA or LMWH alone. The limited number of cases did not allow the determination of the most appropriate dosage of PV-specific therapy, the best combination partner to IFN or the optimal IFN formulation. Other limitations are potential selection bias, as patients with a better pregnancy outcome may have been preferentially enrolled and complicated pregnancies with delivery before PV diagnosis may have been examined in more detail compared with noncomplicated pregnancies. Furthermore, our data derive mainly from tertiary centers, where complicated PV pregnancies are potentially more frequent.

Maternal complications occurred in 22.5% of our cases, which is comparable to previous publications.^3,6^ In this context, it is confirmed that the use of PV-specific therapy did not increase the risk of severe maternal complications according to our regression analysis. Remarkably, the rate of cesarean sections (20.9%) was lower in our study cohort compared with data for pregnancies in healthy women (UK average: 25%^8^; Germany: 29.7% in 2020^14^).

In summary, our analysis has revealed that the live birth rate of PV pregnancies can be significantly improved by the antenatal introduction of PV-specific therapy, particularly ASA in combination with LMWH or IFN (alone or in combination with ASA and/or LMWH), supporting the proposal of Robinson and Harrison.^15^ Importantly, our study also shows that introduction of such PV-specific therapy does not increase the risk of serious maternal or fetal complications.

## AUTHOR CONTRIBUTIONS

KW, MB, and MG did study conception and design. KW, MB, AR, EN-M, MG, SI, SK, TB, FS, KD, J-JK, JB, PS, VK, and TB did data collection. KW, MB, RM, HM, CF, and MG did analysis and interpretation of results. KW and MG did draft article preparation. All authors reviewed the results and approved the final version of the letter.

## DATA AVAILABILITY

The data that support the findings of this study are available from the corresponding author upon reasonable request.

## DISCLOSURES

KW and MG declares funding, advisory board honoraria and other financial support (eg, travel support) from Amgen, AOP Orphan, Novartis, BMS, AbbVie, Pfizer, Roche, Janssen, Gilead, AstraZeneca, Lilly. SI declares honoraria from Pfizer, AOP Orphan, Novartis, Incyte and Abbvie, advisory board honoraria from Pfizer, Novartis, GSK and Incyte and other financial support (travel support) from Pfizer, Novartis, AOP Orphan and Alexion. SK reports funding from Novartis, Bristol-Myers Squibb, Janssen/Geron; advisory board honoraria from Pfizer, Incyte, Ariad, Novartis, AOP Pharma, BMS, Celgene, Geron, Janssen, CTI, Roche, Baxalta, Sanofi, Sierra Oncology, and GSK; patent for BET inhibitor at RWTH Aachen University; honoraria from Novartis, BMS, Celgene, Geron, Janssen, Pfizer, Incyte, Ariad, Shire, Roche, AOP Pharma, Sierra Oncology, Karthos, Imago Bioscience, and GSK; serves as an editor for HemaSphere; and other financial support (eg, travel support) from Alexion, Novartis, BMS, Incyte, Ariad, AOP Pharma, Baxalta, CTI, Pfizer, Sanofi, Celgene, Shire, Janssen, Geron, Abbvie, Karthos, Sierra Oncology, Imago Biosciences, and GSK. KD declares consulting/advisory role/honoraria from AbbVie, Celgene/BMS, Novartis, CTI BioPharma Corp, and Roche. J-JK declares consulting/advisory role/honoraria from AbbVie, BMS, Novartis, AOP Health. All the other authors have no conflicts of interest to disclose.

## SOURCES OF FUNDING

The authors declare no sources of funding for this manuscript.

## Supplementary Material
