# Supplementary material for: Outcome of 129 Pregnancies in Polycythemia Vera Patients: A Report of the European LeukemiaNET
Source: Hemasphere. 2023 May 2;7(5):e882. doi: 10.1097/HS9.0000000000000882 (PMC10155895; doi:10.1097/HS9.0000000000000882)
Supplement: Supplementary file 1 [file hs9-7-e882-s001.docx]

**Table 1: Results from Generalized Linear Mixed Models (GLMMs) of the 129 PV pregnancies in terms of pregnancy outcome.** The 95% confidence intervals for the estimators are given in brackets. Administration of ASA/ LMWH (p=0.001) or IFN (p=0.023) during pregnancy was significantly associated with a lower risk of miscarriage than standard antenatal care.

| **Variable** | **Odds ratios [95% CI]** | **p-values** |
| --- | --- | --- |
| Age at pregnancy establishment | 1.004 [0.882, 1.146] | 0.945 |
| Delivery before or at time of PV diagnosis | 0.731 [0.129, 4.061] | 0.702 |
| ASA monotherapy | 0.692 [0.121, 3.200] | 0.634 |
| LMWH monotherapy | 0.134 [0.010, 0.997] | 0.069 |
| ASA and LMWH | 0.040 [0.004, 0.213] | 0.001* |
| IFN (monotherapy or in combination with ASA and/or LMWH) | 0.079 [0.005, 0.539] | 0.023* |

ASA= acetylsalicylic acid; LMWH= low molecular weight heparin; IFN= interferon-alpha

*statistically significant
