# Supplementary material for: Outcome of 129 Pregnancies in Polycythemia Vera Patients: A Report of the European LeukemiaNET
Source: Hemasphere. 2023 May 2;7(5):e882. doi: 10.1097/HS9.0000000000000882 (PMC10155895; doi:10.1097/HS9.0000000000000882)
Supplement: Supplementary file 2 [file hs9-7-e882-s002.docx]

**Table 2: Results from Generalized Linear Mixed Models (GLMMs) of the 129 PV pregnancies concerning maternal complications.** The 95% confidence intervals for the estimated odds ratios are given in brackets. No significant association was found between these variables and maternal complications.

| **Variable** | **Odds ratios [95% CI]** | **p-values** |
| --- | --- | --- |
| Age at pregnancy establishment | 0.936 [0.823, 1.050] | 0.243 |
| Delivery before or at time of PV establishment | 1.181 [0.191, 9.924] | 0.855 |
| ASA monotherapy | 0.398 [0.041, 2.767] | 0.358 |
| LMWH monotherapy | 0.944 [0.082, 8.040] | 0.957 |
| ASA and LMWH | 1.896 [0.436, 11.710] | 0.402 |
| IFN (monotherapy or in combination with ASA and/or LMWH) | 5.354 [1.025, 56.322] | 0.071 |

ASA= acetylsalicylic acid; LMWH= low molecular weight heparin; IFN= interferon-alpha
